# Bilateral Deep Peroneal Nerve Paralysis Following Kerosene Self-Injection into External Hemorrhoids

**DOI:** 10.1155/2010/850394

**Published:** 2010-09-29

**Authors:** Khalil Rostami, Esmaeil Farzaneh, Hassan Abolhassani

**Affiliations:** ^1^Department of Surgery, Ardabil University of Medical Sciences, Ardabil, Iran; ^2^Department of Medical Toxicology & Forensic Medicine, Ardabil University of Medical Sciences, Ardabil, Iran; ^3^Growth and Development Research Center, Tehran University of Medical Sciences, Tehran, Iran

## Abstract

Along with conventional therapies, some abrogated traditional treatment had been used for hemorrhoids like local Kerosene injection especially for extremely irritated external hemorrhoids. We report a rare case of Kerosene self-injection into the hemorrhoid. Despite antibiotics therapy, extent debridement, and colostomy, the patient died after 24 hours because of heart attack. Moreover, we discuss here the case with contact or injection of hydrocarbon materials and early care action to decrease the extensions of injury and side effects.

## 1. Introduction

Hemorrhoids tissues containing veins were located in the wall of the rectum and anus and may develop inflammation, bleeding, or thrombosis or may become enlarged and protrude outside the canal which, in this case, are called external hemorrhoids [1].

Kerosene and other petroleum oils were used as the drugs of choice for many medical problems in the past years. The most common use of Kerosene was antiseptic aid for cuts and scrapes. Daily applications of Kerosene were also used to treat head lice, athlete's foot, animal problems including cracked or infected hooves, and worm infections [2].

Using a rectal compress saturated with Kerosene still can avail for relief of the pain of extremely irritated external hemorrhoids occurring after childbirth.

Nowadays, it is obvious that Kerosene chemical-based components are toxic materials for the human body. These materials were found to cause significant direct and indirect dangerous effects on health through inhalation, ingestion, injection, or local dermal contacts [3–5]. These negative effects may lead to losing the functions of organs of body or lead to long-term disabilities. Although training and increasing of public awareness about these side effects may prevent self-therapy of Kerosene, early diagnosis of these side effects can help in the results of care. We report a rare case with local and systemic absorbent of Kerosene through hemorrhoidal injection which developed different injuries to the soft-tissue organs and the nervous system.

## 2. Case Report

A 39-year-old man was admitted to the Fatemi Hospital which is affiliated to the Ardabil University of Medical Scinces who was suffering from scrotal pain, scrotal swelling, and urinary retention following repeated self-injection of Kerosene into his protruded external hemorrhoid since previous 6 days. 

The vital signs of the patient were stable in the first visit. In the examination, the bilateral foot drop of the patient was observed in association with reduced plantar flexion and saddle anesthesia which probably showed caudal equine syndrome. In Magnetic Resonance Imaging (MRI), lumbosacral index was normal, but in Electromyography (EMG) and nerve-conducting studies the demyelinization and degeneration of deep peroneal nerve in both knee regions were reported. After preliminary management of the signs and symptoms in the hospital, the patient was released but next appointed visits for the patient were not obeyed. 

One month later, the patient readmitted to the emergency unit of the hospital because of severe extended of necrosis in perineal region and scrotum edema, and he was suggested to under, a surgery of laparotomy and colostomy. This patient had been cared for with antibiotics therapy, washing, debridement, and skin graft during the surgery, but this patient died as a result of massive myocardial infarction, 24 hours after operation.

## 3. Discussion

Kerosene is one of the toxic hydrocarbons to which lower-levels exposures through dermal absorption, pulmonary inhalation, or oral ingestion routes may occur repeatedly for all persons, especially for urban people. Attritional effects on the nervous system, decreased immune system function, autoimmune disorders, asthma, allergies, infertility, miscarriage, and child behavior disorders including learning disabilities, mental retardation, hyperactivity, and attention deficit disorders were described after prolonged and chronic Kerosene exposure, while there is little epidemiological evidence for fuel-induced death, cancer, or other serious organic diseases [2–5]. 

Moreover, some new studies reported acute or persisting biological or health effects and side effects on humans or animals because of the acute exposure to kerosene-based hydrocarbon fuels [6]. 

Patients with hemorrhoids often seek medical attention only after a variety of over-the-counter preparations and modalities have failed. There are several available “conservative" options in medical setting for the treatment of symptomatic hemorrhoids and its complaints including bleeding, irritation and pruritus, and thrombosis. Adding fiber to the diet with a dose of 20 to 30 g/day [7] and supplementation with psyllium for six weeks [8] may be beneficial for patients with bleeding and may relieve pruritus related to fecal soilage by the bulking effect. Hydroxyethylrutoside, an oral micronized flavonoid compound, improves venous tone, microvascular permeability, lymphatic activity, and microcirculatory nutritive flow [9]. Moreover, analgesic creams (0.5 percent nitroglycerin ointment [10] and topical nifedipine [11]), hydrocortisone suppositories, and warm sitz baths for the relaxation of the internal anal sphincter had benefit on irritation and pruritus associated with hemorrhoids [12]. These medications also may provide adequate relief until spontaneous resolution of clot occurs in thrombosed external hemorrhoids with excruciating pain. 

“Minimally invasive" (rubber band ligation, infrared coagulation, bipolar diathermy, laser photocoagulation, sclerotherapy, and cryosurgery) [13] or “surgical techniques" (closed hemorrhoidectomy [14], open hemorrhoidectomy, semi-open technique [15], stapled hemorrhoidectomy [16], lateral internal sphincterotomy [17]) almost used to treat internal hemorrhoids and patients with grade IV internal hemorrhoids and some with grade III who develop thrombosis and strangulated hemorrhoid. These procedures usually require general or spinal anesthesia. However, selected patients may tolerate the procedure with sedation and a local anesthetic.

In this case, we described the patient who had suffered from protruded external hemorrhoid during many years and was recommended traditional self-care use and intoxicated with Kerosene, which created acute major injuries to different organs like skin and soft tissues, as result of each entry pathway by two kinds of side effects [18–21]. First pathway is direct local toxic effects of Kerosene on contacted tissues like soft tissues (skin and subcutaneous) which led to toxic peripheral neuropathy and paralysis of motor nervous system. Second pathway is systematic effect of Kerosene through absorption or injection into the blood vessel. Recent effect can lead to creating necrosis and infection. 

In our case, he had signs and symptoms of both local and systematic effects including urinary retention, feet drop, reciprocal deep peroneal nerve paralysis, delayed necrosis of soft tissue, and infection. 

Neurotoxicity of Kerosene was confirmed in previous studies including ataxia, hypoactivity, and prostration of central nervous system by high inhalation exposures [2–5], but current peripheral neuropathy was not reported to our best knowledge. 

Absorption of kerosene range through the skin has been demonstrated to be fairly rapid, but it was limited to approximately 10%–15% after 24 hours of the applied dose [3]. Although Kerosene penetration will not cause systemic toxicity because of low fluxes of all the components, the absorption of aliphatic components into the skin may be a cause of skin irritation [22]. Therefore, assessing risks of systemic absorption of hydrocarbon components is complex because most of the components are present in the mixture in small quantities (less than 1%) [23]. In case of contact or injection of hydrocarbon materials, the fast care action can decrease the extensions of injury and side effect. If the side effect appears, the washing, debridement, and dressing of wound and maintenance care such as physiotherapy of bodies and using of corticosteroids could decrease the extensions of injury to soft tissues. In this case, delayed admission to hospital and interrupted outpatient cares led to the extension of tissue necrosis and poor management of this case.

##  Conflict of Interests

There is no conflict of interest to report.
